# The Role of Testing and Characterization in the Methodical Development of Sensor-Integrating Bolts for Multi-Axial Force Measurement

**DOI:** 10.3390/s26144415

**Published:** 2026-07-11

**Authors:** Julian Peters, Klaus Rappenecker, Fabian Deeg, Felix Herbst, David Riehl, Dirk Leiacker, Mario Kupnik, Klaus Hofmann, Sven Matthiesen

**Affiliations:** 1 Institute of Product Engineering, Karlsruhe Institute of Technology (KIT), 76131 Karlsruhe, Germany; klaus.rappenecker@partner.kit.edu (K.R.); fabian.deeg@kit.edu (F.D.); 2 Measurement and Sensor Technology Group, Technische Universität Darmstadt, 64283 Darmstadt, Germany; 3 Integrated Electronic Systems Lab, Technische Universität Darmstadt, 64283 Darmstadt, Germany

**Keywords:** machine element, strain gauge, force, integrated measurement, structurally integrated sensor, linearity, hysteresis, error, sensitivity, functional behavior

## Abstract

**Highlights:**

**What are the main findings?**

- The testing and characterization activities helped systematically gain knowledge on the integration of the sensor, the strain gauge application uncertainty, the measurement errors and the sensitivities of the sensor-integrating bolts.
- Thus, the issues can be traced back to their respective origins, namely, the strain gauges, deformation body, and mounting method and how they influence the linearity and hysteresis errors.

**What are the implications of the main findings?**

- The methodical approach led to a solution of a sensor-integrating bolt representing a compromise between multi-axial force measurement with BLE data interface and maintaining the standardized mechanical interfaces, so that they can be used as normal with no restrictions in terms of installation space or tightening tools.
- The complete integration of the measurement chain reduces the mechanical performance within a range of 1.3% to 24.6% depending on the sensor cavity, highlighting the conflict of objectives between mechanical and sensory disciplines. Measures for compensation need to be taken, such as increasing the bolt’s strength.

**Abstract:**

Sensor-integrating bolts offer a promising solution for in situ data acquisition by integrating sensory functions into existing machine elements. The standardized mechanical interfaces and performance must be maintained in order to ensure widespread use and retrofitting, which leads to a conflict of objectives between mechanical and sensory performance. The state of the art lacks multi-axial force-measuring bolts that meet these objectives. Therefore, we present a methodical approach that develops evolving prototypes of sensor-integrating bolts and applies testing activities to characterize the bolts’ performance and gain knowledge to further improve the prototypes. Strain gauges and a specifically developed electronics platform for data acquisition with a BLE interface are used. The prototypes are tested for axial and bending loads. Mechanical and sensory performance indicators are investigated, such as von Mises stress increase, linearity errors, hysteresis errors and sensitivity. The characterization results of the testing activities and the knowledge extracted from each iteration concerning aspects like strain gauge type and position or sensor body integration and their influence on the performance indicators are shown. Overall, the prototypes improved the linearity error from 3.3% to 1% (axial) and 3.7% to 0.1% (bending). The error is below 6% and sensitivities are around 10 µV/V/kN (axial) and 1 µV/V/Nm (bending). The integration of the measurement chain reduces the mechanical performance within a range of 1.3% to 24.6%, underlining the conflict of objectives. Measures for compensating the reduction need to be taken, such as increasing the bolt’s strength. Moreover, the measurements indicate that bending torques are superposed, even in axial load cases, making investigations of the effect on loadability necessary.

## 1. Introduction

In modern industrial environments, the demand for high-quality operational data is steadily increasing, driven by the need to enable predictive maintenance, condition monitoring and digital twins. Capturing this data directly at functionally critical points within a system—in situ—offers significant advantages in terms of measurement data quality and contextual relevance. Standardized machine elements are located at these key positions, making them highly suitable platforms for integrated sensing solutions [1,2]. Embedding sensor functionality within these machine elements enables them to serve as standardized, high-quality sensor nodes (sensor-integrating machine elements—SiMEs).

Bolts are among the most widespread machine elements and usually within the force flow, making them especially rewarding as a sensor node [3]. Furthermore, they are highly standardized in geometry [4,5] and loadability [6,7], providing little design freedom for integrating sensor modules. Sensor-integrating bolts should comply with these standards in order to ensure broad usability. In addition to tensile forces and torsional torque [8], which form the basis for most bolt dimensioning calculations, bending torques also occur during operation [9,10]. Fatigue failure is one of the most common failure modes for bolted connections [11]. This kind of failure occurs at the highest cyclic stress alternations at the notches of the bolt, usually caused by superposed axial and bending loads.

“Self-induced loosening” is a common failure mode regarding bolts, where cyclic forces perpendicular to the bolt’s axis can cause a loss of preload force. This is measurable by small bending torques of the bolt [12]. Hence, the combination of bending torque and axial force measurement is necessary, which is called multi-axial force measurement.

Kirchner et al. present an overview of the current state of research on SiMEs in general and sensor-integrating bolts in collaboration with the authors [2]. Several approaches for sensor-integrating bolts exist (Table 1). However, there is currently no solution featuring multi-axial force measurement combined with the complete integration of the measurement chain. The existing sensor-integrating bolts are categorized using sensor-specific criteria and also the influence of sensor integration on the mechanical characteristics in order to check whether the bolt can be used as before (Table 1).

Most approaches use strain-based sensor principles—either strain gauges (SG) or piezo-based elements—for force measurement. The axial force is most commonly measured in existing solutions; however, for bending torque measurements, more space is needed externally or within the bolt, and therefore these solutions do not fulfill the requirements. Examples include Fraunhofer and Biehl et al. [13,14], where the sensors and electronics are located in a washer and a housing externally, increasing space usage.

In this work, the term multi-axial force measurement, as described in [15,16,17], is used. It is based on three or more measurement axes with co-linear directions.

**Table 1:** State of the art of sensor-integrating bolts based on [2].

Sensor Principle/Location	Measured Physical Quantity	Data & Power Transmission	Change in Mechanical Characteristics	Preservation of Mechanical Interfaces	References
Strain gauges (SG) on bolt head	Uniaxial preload force (static and dynamic), partly with extra SG for temperature compensation	Wireless (RFID)	None	No	[18,19]
3x pressure sensitive thin-film sensor in washer	Axial force and bending torques, temperature	Wireless (LPWAN); solar-powered	None	No	[13,14]
Pressure-sensitive piezoresistive layer on washer	Axial force and bending torques	RFID	None (extra washer)	Washer needed	[20]
3x SG on deformation body in bolt shaft	Uniaxial preload force	Wired connector at bolt head	20% reduction in loadability	Partly (wire)	[21]
Piezo SG in bolt shaft	Uniaxial preload force	Wired plug connector at bolt head	No (compensated)	Partly (wire)	[22]
Multiple SG in shaft cavity, IMU	Multi-axial force, temperature, acceleration, angular speed, orientation	Wireless (BLE); integrated energy storage	Yes	Yes	[23]
SG at thread root	Axial forces	Wired	None	No	[24]
Analog measuring pin at bolt head	Uniaxial preload force (distance between pin and bolt head)	Optical; external wireless electronics	None	No	[25]
Ultrasonic pulse reflection at bolt head	Uniaxial force via longitudinal strain	External, wired electronics	None	No	[26]
Fiber bragg grating optical sensor distributed in shaft	Axial force, torsional torques and temperature for compensation	External, wired optical fibers and electronics	Yes, boreholes in shaft	No	[27,28]
Capacitive sensor under thread	Distance	External, wired measurement electronics	None	No	[29]

To further capitalize on that potential, SiMEs are being researched, e.g., in the priority program “Sensor-integrating machine elements as enabler for widespread digitization” (SPP2305) [30], of which this contribution is a part. A key objective is to maintain the machine element’s mechanical performance while integrating the novel sensory function with a complete measurement chain and achieving high measurement quality. A conflict of objectives between sensory and mechanical domains exists because they share the same design parameters [31]. Sensor modules require space in specific locations that are relevant for measuring the physical quantities, while mechanical integrity depends on preserving material strength and geometry. This interplay becomes especially pronounced in bolts, where cavities for sensor integration can significantly reduce load-bearing capacity [32].

### 1.1. Previous Work

In previous work, the conflict of objectives between mechanical and sensory disciplines is addressed based on a negotiation model, leading to design space identification for the sensors within the bolts [32]. Furthermore, in previous work, a method to support the development of sensor-integrating bolts through a combination of the V-model and the method of testing to acquire missing knowledge was presented [33]. Furthermore, miniaturized electronics for acquiring and communicating sensor signals from within the bolt were developed [34]. This contribution builds upon the design space identification [32] and the high-level method [33] from the previous work. It first shows the design and evaluation of the evolving iterations of prototypes of the sensor-integrating bolts, and then the evaluation results and findings that were obtained and the optimizations that were performed are presented.

### 1.2. Conclusion of the State of the Art and Research Question

The state of the art lacks a sensor-integrating bolt that provides high-quality multi-axial force measurement while maintaining mechanical performance. In previous work, the authors present a solution optimized for bending torque measurement [35] that evolves from the results shown here. The methodical approach and development process have been shown previously [32,33]. However, characterizing the evolving prototypes and learning from each step to optimize the prototypes have not been shown yet. Therefore, the purpose of this contribution is to disclose this insight for use in other sensor integration projects.

The following research question is derived: “How do iteratively designed prototypes of sensor-integrating bolts perform in terms of mechanical and sensory characteristics, and what can be learned for optimization?”

The focus of the characterization will be on the following two aspects:

1. The characterization of the sensor module integrated into the bolts in terms of linearity and hysteresis errors, as well as sensitivity.
2. The influence of the sensor module on the mechanical performance in terms of von Mises stress increase.

## 2. Materials and Methods

### 2.1. Methodical Approach for Development of Sensor-Integrating Bolts

The methodical approach for developing sensor-integrating bolts for multi-axial force measurement is presented in previous work [33]. A combination of the V-Model from VDI 2206 [36] and the methodology of testing is used for guidance and to gather missing knowledge for development. The gathering of knowledge is based on hypotheses that connect functional behavior with the embodiment. Hypotheses are formulated following the structure:

“if *embodiment,* then *functional behavior,* because *connecting relationship*”. 

In the present contribution, we present the prototypes in several evolving iterations that were designed, manufactured and evaluated for their mechanical and sensory performance. We also describe selected hypotheses used for learning from the evaluation.

**Figure 1:**
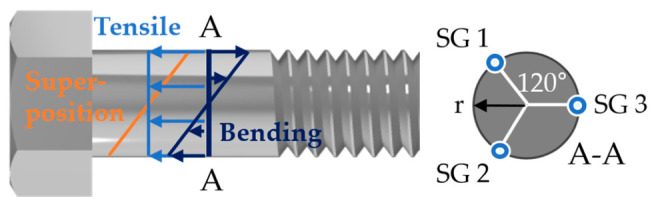
Sensor concept for the multi-axial measurement of tensile and bending strains that occur simultaneously within the bolt. The sensor elements consist of three strain gauges (SGs) aligned coaxially along the bolt axis, which are evenly distributed around the circumference at a constant radius r.

Starting from the analysis of a standardized bolt (M20 × 100 with shaft, ISO 4014 [5]) as a reference, the effect of cavities on the performance when subjected to multi-axial load is analyzed (Figure 1). In parallel, existing sensor-integrating bolts and sensor concepts for the multi-axial measurement of forces are studied, based on which the first iteration, V1, is designed, as presented in Section 3.1. In order to characterize the prototypes, criteria (Section 2.2) and a testing environment are set up (Section 2.3).

The following equations describe how the axial force and bending torque vector can be computed from the three strain gauge signals under linear–elastic conditions. They are based on Rappenecker et al. [12]. The approach determines the maximum strain at the bolt shaft. For this purpose, a geometric model is formulated in which the positions and measured strains of the strain gauges define a plane in an internal bolt coordinate system. This plane is intersected with an idealized cylindrical surface at the radius of the stress cross section (bolt shaft), from which the maximum strain $\varepsilon_{SAb}$ can be calculated (Equation (1)):

(1) $$\varepsilon_{SAb}=z_{max}=\frac{d+r\sqrt{a^{2}+b^{2}}}{c}.$$

The parameters a,b,c,d are constants of the plane equation derived from the positions of the strain gauges and the measured strain values, and r is the radius of the stress cross section or shaft. To determine the bending component of the strain $\varepsilon_{b}$, the purely axial component $\varepsilon_{SA}$, calculated as the mean value of the three strain gauge strains $\varepsilon_{SGi_{SAb}}$, is subtracted from the maximum strain $\varepsilon_{SAb}$ (Equations (2) and (3)):

(2) $$\varepsilon_{b}=\varepsilon_{SAb}-\varepsilon_{SA}.$$

(3) $$\varepsilon_{SA}=\frac{\varepsilon_{SG1_{SAb}}+\varepsilon_{SG2_{SAb}}+\varepsilon_{SG3_{SAb}}}{3}.$$

The strain at the strain gauge positions $\varepsilon_{SGi_{SAb}}$ is calculated from the measured voltage of the Wheatstone bridge, with B denoting the bridge factor, $U_{A}$ the bridge output voltage, and $U_{B}$ the bridge excitation voltage, as follows (Equation (4)) [37]:

(4) $$\varepsilon_{SGi_{SAb}}=\frac{1}{B}\frac{4}{k}\frac{U_{A}}{U_{B}}.$$

The bridge factor B depends on the orientation and wiring of the strain gauges. For the half-bridges used here, it is 1+ν (including transverse contraction) or 2, depending on the grid orientation.

The bending moment can then be calculated from the bending component of the strain $\varepsilon_{b}$, Young’s modulus E, and the section modulus W of the bolt shaft (Equation (5)):

(5) $$M_{b}=\varepsilon_{b}EW.$$

### 2.2. Criteria for Characterizing the Iterations of Sensor-Integrating Bolts and Data Postprocessing

#### 2.2.1. Characterizing the Mechanical Performance

The state of the art describes two common modes of failure: cracks in the lowest cross section for static loads and the highest stress for dynamic loads [38]. These two measures are evaluated in the scope of this contribution. The lowest cross section is calculated by geometry that is explained in the design sections.

The von Mises stress is established as an indicator if subjected to multi-axial loading [31,39]. It combines tensile, bending and torsional stress in one value. In previous work, a FE-model has been set up that calculates a relative increase in the maximum von Mises stress due to cavities of varying sizes in an M20 bolt [32]. This von Mises stress increase is used as another criterion for characterizing the mechanical performance.

#### 2.2.2. Characterizing the Sensory Performance

Regarding the **sensory performance**, the errors in linearity and hysteresis as well as the sensitivities are analyzed for static loads. Therefore, the bolts are subjected to a loading pattern in the axial and bending direction based on DIN EN ISO 376 [40]. For the linearity error ($e_{lin}$), a best fit of the strain gauge signals with respect to the reference force sensor is calculated using linear least squares. The distance between the mean of all points within one load step to the fit line for the same reference force represents the linearity error and is calculated in percent as

(6) $$e_{lin}=100\frac{\max\left(\mathrm{fit}\;-\frac{\sum_{i}^{n}sensor_{step,i}}{n}\right)}{\max\left(sensor\right)}.$$

The distance between the points of the upslope and the points of the downslope for the same reference force represents the hysteresis error ($e_{hyst}$), which is calculated in percent as

(7) $$e_{hyst}=100\frac{\max\left(\frac{\sum_{i}^{n}sensor_{step,up,i}}{n}\;-\;\frac{\sum_{i}^{n}sensor_{step,down,i}}{n}\right)}{\max\left(sensor\right)}.$$

This results in a maximum linearity and hysteresis error per strain gauge. For comparison, each sensor is characterized by the largest error only.

The sensitivity is calculated by the gradient of the linear best fit line. The unit of the strain gauge sensor signals is given as output per excitation voltage in V/V. Thus, the unit of the sensitivity s is V/V/N. The minimal force quantization is calculated using the sensitivity and the quantization voltage of the ADC (see Section 2.4).

(8) $$F_{n,min}=\frac{V_{quant}}{S\;V_{bridge}}=\frac{V_{ref}2^{\left(ENOB\right)}}{S\;V_{bridge}}.$$

All postprocessing is performed with MATLAB R2024b (MathWorks Inc., Natick, MA, USA). All data except test run V3 are set to zero prior to the loading and are median-filtered with a window size of five to reduce noise.

### 2.3. Testing Environment

**Figure 2:**
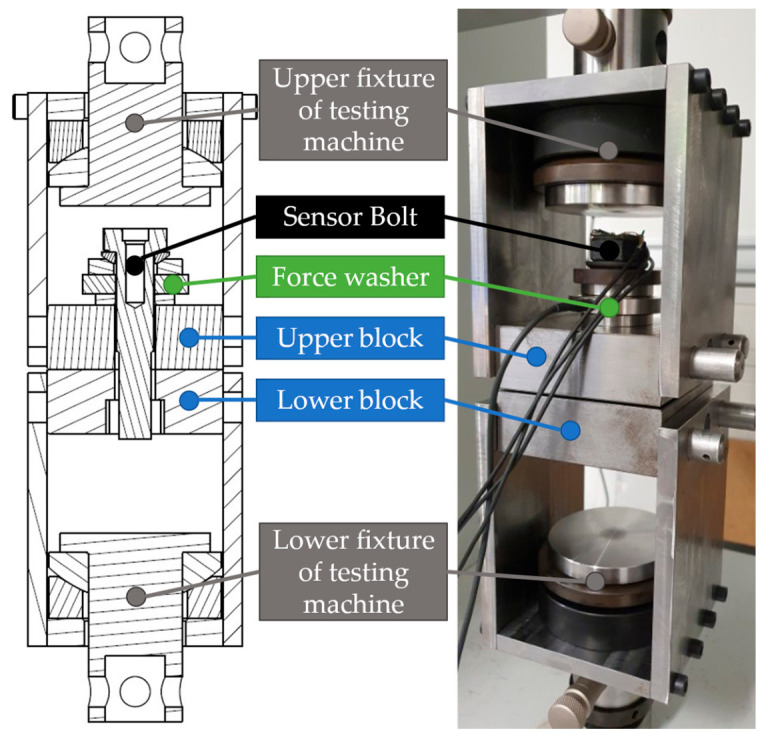
Setup in universal testing machine, consisting of two blocks clamped by the sensor-integrating bolt and a force measuring washer in between. The technical drawing (**left**) shows the assembly.

The testing environment is a universal testing machine for push or pull force excitation (112-50 kN TesT GmbH, Solingen, Germany). The machine has a reference force sensor (301-50 kN, class 0.5, TesT GmbH, Solingen, Germany) and an integrated incremental displacement sensor with a resolution of 1 µm. For axial force excitation, two blocks (42CrMo4 steel) are used, which are clamped by the sensor-integrating M20 threaded bolt under investigation (Figure 2). One block has a hex nut cutout that either the nut or the head of the bolt fits into for easier tightening. A force-measuring washer (KR20plus, HBM, Darmstadt, Germany) is mounted between the head and upper block as an additional reference sensor (only used for tightening, otherwise the force sensor of the universal testing machine is used, Figure 2). For the tightening test case, the same testing environment is used, however the load is introduced by a torque wrench.

**Figure 3:**
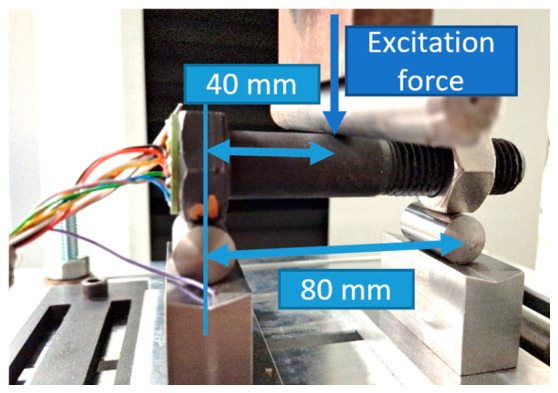
Three-point bending setup for bending torque measurement characterization.

In this study, the load excitations axial force and bending torques are considered separately, as this allows a distinct characterization and is a necessary step towards a multi-axial loading. In theory the strains should superimpose as shown in Figure 1 and Equations (1) to (5). The bending part can be separated by subtracting the mean of the three strain gauges, which corresponds to the axial part [12]. Multi-axial loading is not considered in this study because the testing machine is not able to take a multi-axial reference measurement. The bending torque measurement characterization is a three-point bending setup based on ISO 7438 [41], which is used within the universal testing machine to ensure pure bending load (see Figure 3). The bolt’s head and a nut screwed onto the thread are placed on support cylinders with a distance of 80 mm, and the force excitation is introduced in the middle of the support via another cylinder. The nut is secured on the bolt’s thread using thread glue to avoid unintentional turning. Alignment in the bolt’s axis is checked visually with a gauge.

### 2.4. Data Acquisition and Postprocessing

Data acquisition is performed with a state-of-the-art strain gauge amplifier and acquisition system (GSV4-USB, ME-Systeme, Hennigsdorf, Germany) and, for comparison in one test run, also with the specifically developed “Flexible Ultra Low Power Strain Gauge Readout Platform” (F^2^LECS), published in previous work [34,42]. This allows for the miniaturized electronics’ effect on the measurements to be investigated and compared with a state-of-the-art acquisition system.

**Figure 4:**
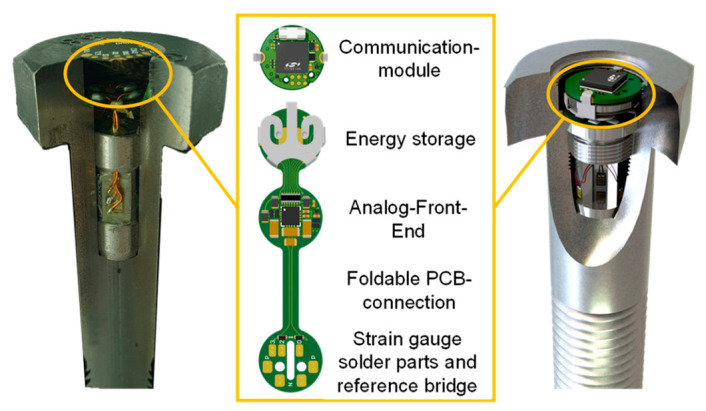
Integrated flexible, modular electronics platform for acquisition and communication of strain gauge signals that is foldable into the bolt’s head, based on [34].

F^2^LECS is a modular platform for sampling and communicating the strain gauge signals. It was developed to be integrated into the M20 bolt’s head and therefore measures only 13 mm in diameter. It consists of a flexible, foldable part with soldering pads for three half-bridge strain gauges, one reference bridge, 2.5 V bridge supply and the analog-front-end ADS1220 from Texas Instruments (Dallas, TX, USA) with a resolution of 24 bits (Figure 4). This results in 149 nV quantization steps per bit. The modular design enables the use of different communication and energy harvesting platforms. Within this contribution, Bluetooth Low Energy is used. The system is designed for minimal energy consumption per measurement and is able to compensate temperature-dependent offset drift by inverting the strain gauge bridge supply [34].

The bolts are subjected to a loading pattern in the axial and bending direction based on DIN EN ISO 376 [40] using three preloading cycles and a minimum of five equidistantly spaced loading steps (including zero) in three repetitions (exception for tightening, only four steps due to the limitations of the tool). The preloading pattern is disregarded in further analysis. Only the load stepping pattern in three repetitions is analyzed. For each loading step, the mean of the strain gauge signals is calculated, disregarding the first and the last 20% of datapoints of each step to keep the control behavior of the testing machine out of the results. The linearity and hysteresis errors, as well as the sensitivities, are calculated as defined in Section 2.2.2.

### 2.5. Test Cases

The iterations of the sensor-integrating bolts (Vi) are characterized using the aforementioned testing environment and applying the following test cases. An exception is the last iteration (V3), for which the results published in [35,43] are used.

A mapping of test cases to the bolt iterations is shown in Table 2.

The maximum force excitations are limited to prevent damaging the bolts. For the axial test case, a typical measurement case is considered: the preload force. This is 76 kN to 100 kN for original M20 bolts (without sensor cavities) depending on the friction (VDI 2230) [7]. Reduced by means of the cavity (maximum of a 24.6% stress increase, which can be transferred to force excitation; see Table 3 and [32]) for the sensors and electronics, this decreases to between 57.3 kN and 75.4 kN. Hence, 50 kN is chosen for axial excitation.

For bending excitation, the maximum torque $M_{b}$ is calculated using 90% of the maximum stress σ, as suggested by VDI 2230; the moment of inertia *I*; and the distance to the outer fiber $e_{max}$:

(9) $$M_{b}=\frac{0.9\;\sigma_{max}\;I}{e_{max}}\;=\;\frac{0.9\cdot 640\;Nmm^{-2}\cdot 4004.2\;mm^{4}.}{10\;mm}\;=\;230.6\;Nm.$$

In order to have a safety margin before plastic deformation occurs, a maximum bending excitation of 200 Nm is chosen.

## 3. Design, Characterization and Learning

In this section, the evolving iterations of the sensor-integrating bolts are presented with the results of characterization, the learning and derived optimizations.

### 3.1. V1: Cylindrical Strain Gauges in Shaft

#### 3.1.1. Design

**Figure 5:**
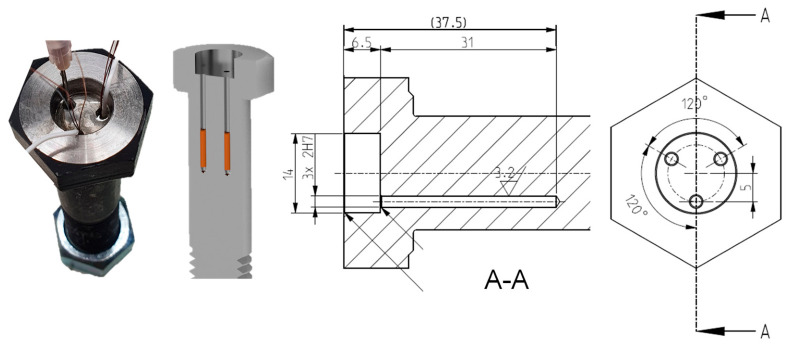
Sensor-integrating bolt V1 as manufactured (**left**), rendering showing position of two of the three strain gauges (**middle**) and technical drawing.

Multi-axial force measurement, in this case measuring axial force and bending torques, is shown to work with measuring the deformation in three or four axes [15,44]. The company HBM (Darmstadt, Germany) offers cylindrical strain gauges for bolts that can be glued into cavities with a diameter of 2 mm. As a first concept, three of these strain gauges are applied into cavities in the shaft of a bolt, combined with another cavity in the head for integrating electronics to acquire the strain gauge signals (Figure 5). The multi-axial measurement, in this case measuring axial force and bending torques, is based on a combination of the approaches of Noh et al. [44] and Baki et al. [15] using strain gauges as a T-rosette half-bridge per axis.

V1 has the following specifications:

- Sensor elements: Three cylindrical strain gauges in T-rosette package as half-bridge, resistance 1 kΩ (TB21, HBM, Darmstadt, Germany);
- Remaining cross section at sensor elements 307.4 mm^2^ (97% of shaft area);
- Sensor arrangements: Collinear to bolt’s main axis, distance of 5 mm to central axis, spacing 120°;
- Adhesive: EP70 (recommended adhesive for this type of strain gauge from HBM, Darmstadt, Germany), maximum five to ten load cycles at maximum strain;
- Electronics cavity: In bolt’s head—diameter 14 mm, depth 6.5 mm.

#### 3.1.2. Characterization and Learning

In terms of the mechanical performance, V1 shows an increase of only 1.26% of the von Mises stress. The cross section’s area is only reduced by 3%.

**Figure 6:**
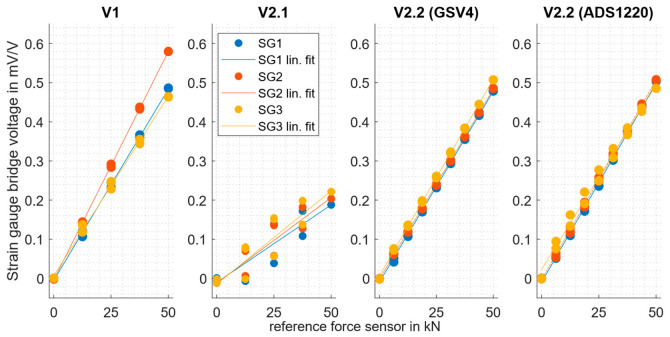
Test case 1 central axial excitation: Linear fit plots. The strain gauge signals are shown with respect to the reference force signal of the excitation. V1 and V2.2 have similar performance, but V1 differs in the SG sensitivities, which can be caused by a superposed bending torque or a faulty SG. V2.1 shows a large hysteresis error and a low sensitivity (gradient), indicating a failure of the interference fit of the sensor body or strain gauge application. V2.2 with F^2^LECS shows similar performance and slightly higher errors for SG3 compared to the state-of-the-art strain gauge amplifier GSV4.

In order to characterize the sensory performance, the bolt was excited with the force pattern according to Section 2.4 in the axial and bending direction. The timeseries plots of axial excitation are shown in Appendix A.1. Figure 6 shows the linear fit plots of V1 in order to determine the sensitivity, linearity and hysteresis errors for **axial direction**. The maximum linearity and hysteresis errors are below 4%, and the mean sensitivity is 10 µV/V/kN (Table 3). With the data of F^2^LECS (Section 2.4 and Formula (3)), the axial force resolution under ideal conditions is 5.96 N. The sensitivity of one strain gauge (SG2) is higher than the other two, indicating a superposed bending torque, even though the excitation is purely axial. This may either be explained by application issues of the strain gauges or by tolerances and irregularities in the surfaces of the setup in the universal testing machine. Application issues can be ruled out, because the pattern changes with the rotation of the bolt relative to the clamped parts.

The tightening test case (TC2) indicates a stable and repeatable strain gauge signal for the three cycles of tightening and loosening. The characteristics are similar to TC1: the maximum linearity error is 3.7%, and the sensitivity is 10.6 µV/V/kN (see Table 3).

**Figure 7:**
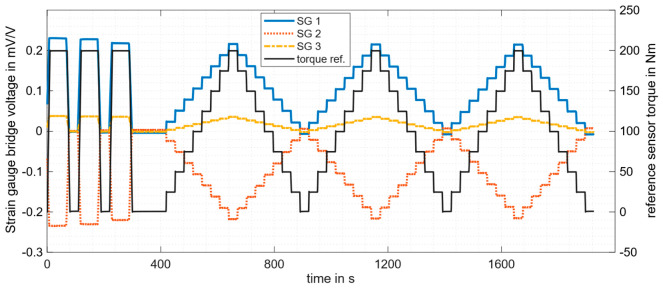
Test case 3 with V1: timeseries plot of 3-point bending with excitation direction 331° with respect to SG1; SG signals show positive and negative courses depending on whether they are in the positive or negative strain part of the bolt, which is expected behavior for bending excitations. The reference torque is calculated from the reference force sensor values and geometry of the setup (Section 2.4).

**Figure 8:**
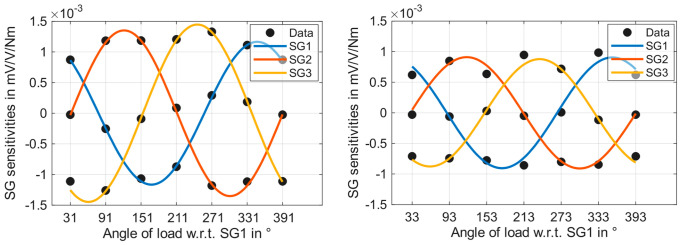
Test case 3: Sine fit plot of the sensitivities with respect to the rotating excitation direction (zero at SG1) for V1 (**left**), V2.2 (**right**). The three sine curves are spaced relative to each other in the same way as the SGs: 120°.

Concerning the **bending direction**, the strain gauge signals show positive or negative values depending on their location in the positive or negative strain area, meaning the upper and lower half of the bolt (Figure 7). A value close to zero would mean that the SG is in the neutral phase of the bending (SG3, Figure 7). The three repetitions show a repeatable performance. Sensitivities for each strain gauge and excitation direction are calculated based on the linear fits (Appendix A.3). The sensitivities depend on the direction of the excitation with respect to the location of the strain gauges, measured after drilling of the boreholes with an angular measurement gauge. This is depicted in the sine fit plot of the sensitivities (Figure 8): each of the three strain gauge signals follows a sine with a change in the excitation direction and the signals shift toward each other by the angle between the strain gauges of 120°. This shows a correct behavior of the measurement signal for bending measurements. Thus, by using the sine fit curves, the angle of the load excitation can be retrieved. For characterization, the maximum sensitivities and mean errors of all angles are taken. The maximum errors for bending are 3.68% (linearity) and 2.53% (hysteresis). The maximum sensitivity is 1.3 µV/V/Nm. This results in a torque resolution of 46 mNm for pure bending load in ideal conditions with data of F^2^LECS (24 bit ADC, 149 nV resolution, 2.5 V bridge supply).

A problem with the application of the cylindrical strain gauges is the impossibility to apply pressure during the curing of the adhesive. This leads to a rather thick layer of adhesive between the strain gauge and the bolt, which cannot be controlled repetitively. Due to this, the strain gauge may not follow the strain of the bolt repetitively. Hence, this setup is likely prone to uncertainty, especially with the influence of temperature changes on the adhesive layer. Furthermore, the recommended adhesive is only designed for five to ten load cycles according to the datasheet from the manufacturer HBM. The issue of the limited load cycles and the application of pressure while curing the adhesive is solved by the next iteration V2.1.

### 3.2. V2.1: Flat Strain Gauges on Sensor Body

#### 3.2.1. Design

Transformation of the previous problem into a new design: The problem with the previous version is the uncertainty of the adhesive layer due to the limited ability to apply pressure during curing and visual inspection. Pressure on the cylindrical strain gauges can only be applied using an inflatable balloon, and visual inspection would still be impossible. Another option is flat strain gauges. To apply pressure and enable visual inspection, the gauges need to be applied on the outside surfaces of a distinct sensor body, which is mounted into the bolt after strain gauge application.

Hypothesis: If the strain gauges are flat and bonded on a distinct sensor body, then the uncertainties caused by the adhesive can be reduced, because the adhesive layer can be controlled and inspected.

**Figure 9:**
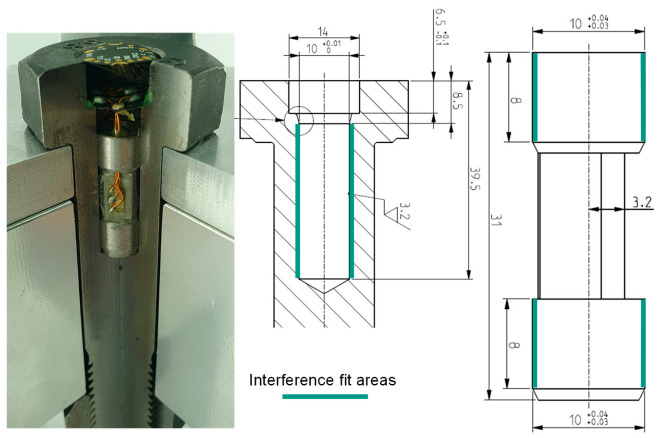
Sensor-integrating bolt V2.1 as manufactured and cut open for demonstration (**left**), technical drawings of bolt (**middle**) and sensor body (**right**) showing interference fit.

V2.1 is depicted in Figure 9. It has the following specifications:

- Sensor elements: Three cylindrical strain gauges in the T-rosette package as half-bridge, resistance 1 kΩ (N5K-06-S5045H, Micro-Measurements supplied via ME-Meßsysteme GmbH, Hennigsdorf, Germany).
- Remaining cross section at sensor elements:
  $\pi (R^{2}-r^{2})+A_{Sensor-body}=[\pi (10^{2}-5^{2})+49.47]\;{mm}^{2}=285.1\;{mm}^{2}$ (91% of shaft area).
- Sensor arrangements: On sensor body, co-linear to bolt’s main axis, distance of 3.2 mm to central axis, spacing 120°.
- Adhesive: X60 (HBM, Darmstadt, Germany).
- Sensor body force transmission: The axial force splits up between the sensor body and the rest of the bolt in proportions of their cross section, which results in a factor of $49.47\;{mm}^{2}/285.1\;{mm}^{2}=0.17$ for the sensor body. For 50 kN force excitation (the maximum of the testing machine), this requires an axial force transmission of 8.5 kN on the sensor body.
- Sensor body interference fit: Diameter 10 mm, tolerances +40 µm maximal, +30 µm minimal, Young’s modulus 210,000 N/mm^2^, roughness 3.2, friction 0.15, yield strength 640 N/mm^2^ that results in an axial force transmission capability of 11.1 kN (maximal), 5.18 kN (minimal) and 8.14 kN (mean), using formulas from [45] (pp. 130–131). This is close to the requirement; with regard to the tolerances, the real transmittable force will vary.
- Electronics cavity: Same as V1.

#### 3.2.2. Characterization and Learning

In terms of mechanical performance, V2.1 shows an increase of 18.4% in the von Mises stress [32]. The cross section is reduced by 9%.

**Figure 10:**
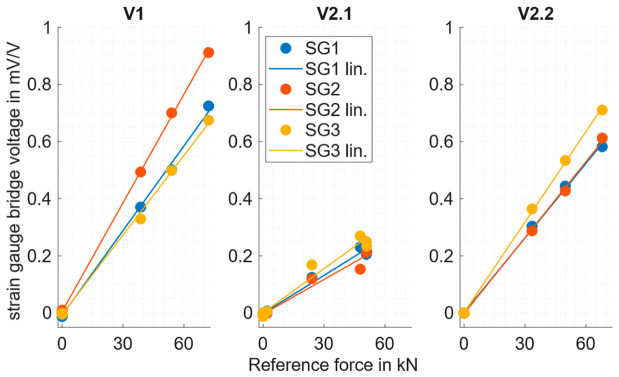
Test case 2 tightening: Linear fit plots. The strain gauge signals are shown with respect to the reference force signal of the excitation. V1 and V2.2 have similar performance with V2.2 showing slightly lower sensitivity. As seen in TC1, V1 has a bigger spread of sensitivities among the three strain gauges and V2.1 shows a large hysteresis error as well as low sensitivity (gradient), indicating a failure of the interference fit of the sensor body or strain gauge application.

In order to characterize the sensory performance, the bolt was excited with the force pattern as shown before with V1 in the axial and bending directions. The timeseries plots are shown in the Appendices. Figure 6 shows the linear fit plots of V2.1 for the **axial direction**. It is obvious that the linearity and hysteresis errors as well as sensitivity are worse compared to V1 (see Table 3). This is also observable for TC2 (Figure 10). In TC1, the hysteresis starts at a reference force of approx. 25 kN. This can be caused by the interference fit failure of the sensor body, localized plastic deformation or damage to the strain gauges. It is assumed that the interference fit failure is the most likely cause, where the sensor body does not follow the strain on high forces and instead slides back. With decreasing loads, the linear fit plot shows an inverse behavior. This hints to insufficient pressure that is not enough to transmit the forces. In addition, the continuous fit surface within the bolt could have been damaged during press fitting of the sensor body. The **bending direction** was aborted due to the failure of the interference fit. In the next iteration, the interference fit needs to be redesigned in order to transmit the forces correctly and prevent damage during press fitting, as described in the following section.

Although failure of the interference fit is considered the most likely cause, the present data do not allow for complete separation from secondary damage mechanisms. Local plastic deformation of the mating surfaces could reduce the contact pressure and promote slip, while damage to the strain gauge grid or the adhesive layer could reduce strain transfer and contribute to the observed hysteresis and signal decrease under constant load. The bridge resistances were checked and remained at the expected value of 1 kOhm, which does not indicate an electrical failure of the strain gauges. Further verification could be achieved by targeted push-out tests after loading, repeated zero-signal and drift checks, and microscopy-based inspection of specifically prepared cross sections. Therefore, V2.1 is interpreted as an interference-fit-dominated failure, with possible secondary contributions from plastic deformation or sensor/adhesive damage.

### 3.3. V2.2: Flat Strain Gauges on Hollow Sensor Body

#### 3.3.1. Design

Transformation of the previous problem into the new design: The problem with the previous version is the failure of the press fit. To increase the transmittable forces, the interference can be increased. This is not possible in this case because the limit of surface pressure in the bolt’s fit surface has already been reached. Another option is the reduction in the tolerance fields for manufacturing to prevent unfavorable combinations of the interference measures. However, the available manufacturing has also reached limit with the current tolerance fields. Thus, the remaining option is to decrease the force that the interference fit needs to transmit by increasing the sensor body’s elasticity. This is achieved by using a central borehole, thus preventing a decrease in the radius of the strain gauges’ position, which is important for bending torque measurement sensitivity. Furthermore, the upper and lower fit surfaces of the sensor body are separated by increasing the diameter of the upper fit by 1 mm. This prevents the surfaces from being scraped by mounting, and thus prevents flattening.

Hypothesis: If the elasticity of the sensor body is increased, then the force to be transmitted is decreased, because the ratio is dependent on the elasticity. The success of this method can be evaluated by hysteresis error.

**Figure 11:**
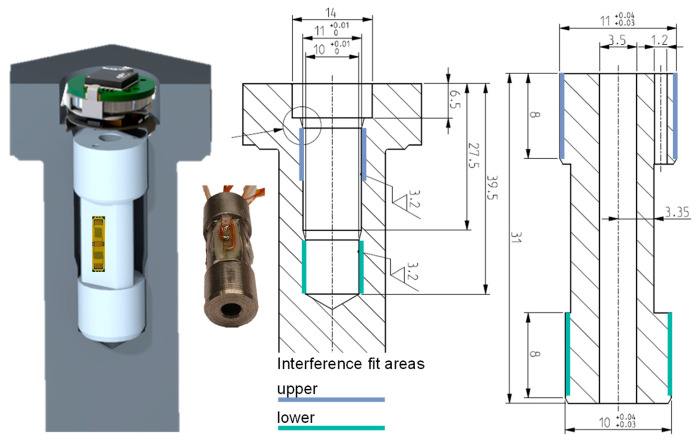
Sensor-integrating bolt V2.2 in rendering and manufactured sensor body (**left**), technical drawings of bolt (**middle**) and sensor body (**right**) showing interference fit with separated areas for upper and lower part to prevent damage during press fitting.

V2.2 is depicted in Figure 11. The specifications are as follows:

- Sensor elements: Same as V2.1.
- Sensor arrangements: Same as V2.1, except the sensor body has a borehole of 3.5 mm and the fit surfaces are separated, and the upper surface diameter is increased to 11 mm. Additionally, the distance between the strain gauge and the central axis is increased to 3.35 mm in order to obtain a higher signal.
- Remaining cross section at sensor elements:
  $\pi (R^{2}-r^{2})+A_{Sensor-body}=[\pi (10^{2}-5.5^{2})+10.99]\;{mm}^{2}=230.11\;{mm}^{2}$ (73% of shaft area).
- Adhesive: Same as V2.1.
- Sensor body force transmission: The axial force splits up between the sensor body and the rest of the bolt in proportions of their cross section, which results in a factor of $10.99\;{mm}^{2}/230.11\;{mm}^{2}=0.048$ for the sensor body. For 50 kN force excitation (maximum of the testing machine), this requires an axial force transmission of 2.39 kN on the sensor body.
- Sensor body interference fit: Using the same interference as V2.1 (diameter 10 mm and 11 mm, +40 µm maximal, +30 µm), this results in an axial force transmission capability of 10.1 kN (maximal) and 4.69 kN (minimal), using formulas from [45] (pp. 130–131) (the reduction compared to V1 is due to the hollow sensor body). Now, the minimal interference will be sufficient to transmit the required force. Moreover, higher force excitations up to 100 kN are possible for minimal interference.
- Electronics cavity: Same as V2.1.

#### 3.3.2. Characterization and Learning

In terms of the mechanical performance, V2.2 shows an increase of 18.4% of the von Mises stress [32]. The cross section is reduced by 27%.

The linear fit plot for V2.2 proves the success of the iteration for the sensory function (Figure 6). Increasing the sensor body’s elasticity led to a sufficient force transmission. In the **axial excitation test case (TC1)**, the sensitivity is similar to V1 and the linearity and hysteresis errors are at 2.2%, which is lower than V1 (see Table 3). The fit plot shows that there is no longer any superposed bending torque in the axial excitation test case (Figure 6), as seen in V1.

The **tightening test case (TC2)** shows similar errors and sensitivities compared to TC1. V2.2 has a lower spread of the sensitivities among the three strain gauges compared to V1 and improves the hysteresis error as well as the sensitivity compared to V2.1. The timeseries plots can be found in Appendix A.2.

Concerning the **bending direction (TC3)**, Figure 8 shows the course of the sensitivities over rotating bending loads, which follow a sine with respect to the excitation direction, comparable to V1. However, the sensitivities are about 0.5 µV/V/Nm smaller than V1, which is approximately 66%. The reason is the strain gauge’s offset to the central axis of the bolt. For bending excitation, the strain increases linearly with rising offset. In V1, the strain gauges have an offset of 5 mm to the central axis; in V2.2, the offset is 3.35 mm, which is approximately 67%. This matches the difference in the sensitivities. The maximum errors for bending are close to V1: 3.67% (linearity) and 2.73% (hysteresis). The fit of the sine curve on the measurements is better for V1.

In order to achieve a better bending torque measurement sensitivity, the distance between the strain gauges and the central bolt axis needs to be increased. However, this is not possible without further harming the mechanical performance. Increasing the distance requires an increase in the sensor body’s diameter. In order to guarantee the interference fit of the sensor body, the borehole in the sensor body must be increased as well, which reduces the minimal cross section and thus the force the bolt can bear. A new design of the sensor body is needed to further increase the sensitivity, especially for bending torques.

With V2.2, the novel F^2^LECS platform is used next to the state-of-the-art GSV4 in order to investigate its influence on the data acquisition. The errors and sensitivities are in a similar range, increased by 2.6% (linearity) and 3.8% (hysteresis) (Table 3). Only SG3 shows slightly higher errors compared to the GSV4. That means F^2^LECS has only a small impact on the characteristics.

### 3.4. V3: Flat Strain Gauges on Bending Optimized Sensor Body

#### 3.4.1. Design

Transformation of the previous problem into a new design: The problem is the decreased sensitivity for bending torque measurement. A new sensor body design is needed for improvement, because the current design is at its limit regarding the distance of the strain gauges to the center axis in combination with the Wheatstone bridge factor, influenced by the T-rosette strain gauge configuration.

Hypothesis: If the strain gauges are applied on bending beams inside the bolt, then the sensitivity for bending increases, because the Wheatstone bridge factor for bending is increased.

**Figure 12:**
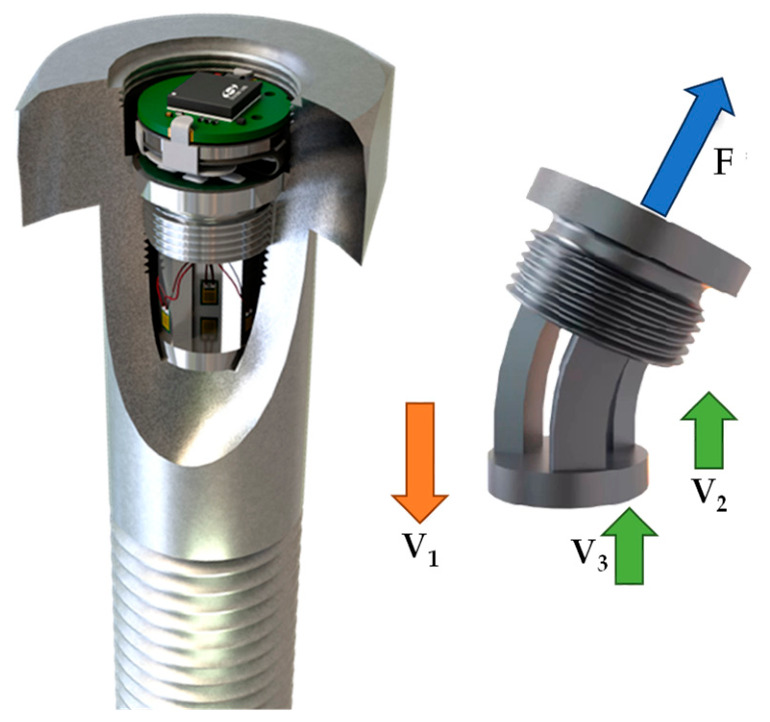
Sensor-integrating bolt V3 (**left**) and sensor body with bending beams (**right**) showing the potentials of the half-bridge strain gauges of each bending beam when a bending torque is applied globally; based on [35].

The new design is presented in [35], depicted in Figure 12. Three beams are regularly spaced off-center, and each has a strain gauge applied on the outside and inside, configured in a half-bridge setup to measure the bending of each beam (Figure 12, left). When bent, all of the beams will bend in the same direction globally, leading to a shift in the potentials of the half-bridge strain gauge outputs per bending beam, as shown in Figure 12 (right). One output will have the opposite sign from the others. For axial excitations, the behavior is different: the half-bridge strain gauges on each beam are configured in bending setup, canceling out the axial elongation. Hence, the axial force cannot be measured in that way. However, when excited with axial load, all of the beams will also bend due to their distance to the central axis. All half-bridge strain gauge outputs will have the same sign. For this reason, the design offers separate sensitivities for axial and bending excitation, because the bending caused by the axial force is lower than the bending caused by bending excitation.

Mounting of the sensor body is realized by a thread on the upper part and adhesive on the lower part.

V3 has the following specifications:

- Sensor elements: Six flat strain gauges, configured as half-bridges per beam for bending measurement, resistance 1 kΩ (N5K-06-S5094Q-10C, ME-Meßsysteme GmbH, Hennigsdorf, Germany).
- Remaining cross section at sensor elements:
  $\pi (R^{2}-r^{2})+A_{Sensor-body}=[\pi (10^{2}-6^{2})+18]\;{mm}^{2}=219.06\;{mm}^{2}$ (70% of shaft area).
- Sensor arrangements: Collinear to bolt’s main axis, circular spacing 120°.
- Adhesive: Hot-curing phenolic resin (P250, HBM, Darmstadt, Germany).
- Electronics cavity: In bolt’s head—diameter 16.4 mm, depth 6.4 mm.

#### 3.4.2. Characterization and Learning

In terms of the mechanical performance, V3 shows an increase of 24.6% of the von Mises stress according to [32], and based on another FE-model an increase of 23.5% according to [35]. The cross section is reduced by 30% (Table 3).

The sensory characteristics are published in [35]. The axial linearity error is 1%, which is the best of all versions. The hysteresis error is 6.15%. The bending linearity and hysteresis errors were evaluated on the sensor body only, not within the bolt. They are lower than the other versions at 0.11% and 0.52%.

### 3.5. Summary and Comparison of the Characteristics

**Figure 13:**
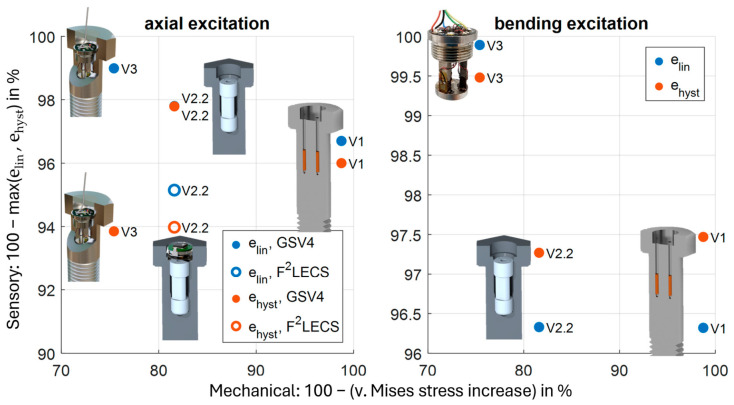
Comparison of mechanical and sensory characteristics. The highest performance is in the upper right corner. V3 has the best sensory performance in terms of the linearity error for both axial and bending excitation, but also the lowest mechanical performance (highest stress increase). V1 has the best mechanical performance, but also the lowest sensory performance in terms of the linearity error. This shows the conflict of objectives between good mechanical versus good sensory performance, which is especially pronounced in the axial direction for V1, V2.2 and V3 and in the bending direction for V1 and V3. The miniaturized low power electronics F^2^LECS worsens the errors slightly compared to the state-of-the-art GSV4.

In this section, the results of the characterization for V1-V3 are compared. The data is summarized in Table 3 and visualized in Figure 13. The individual strain gauge data for each test case is provided in Appendix A.1 and Appendix A.2. All iterations show errors close to or lower than 6%, except for V2.1 due to the failing interference fit. The sensitivities of V1 and V2.2 are similar in the axial direction, while V1 has higher sensitivity than V2.2 in the bending direction. This means that V1 is already a good solution if the uncertainty due to the strain gauge application, especially when subjected to temperature changes, is not an issue. Temperature dependency was not investigated. V1 has the advantage of easy manufacturing, because strain gauge application is easier compared to the other iterations and no sensor body is needed. V2.1 reduces the uncertainty in strain gauge application by using a sensor body, but has a failing interference fit at high forces. V2.2 improves the design of the sensor body for the interference fit, as shown by lower errors. The errors in the axial direction are even lower than V1; however, the sensitivity in the bending direction is also lower due to the radius of the strain gauges to the central axis. This cannot be improved with the V2.2 design of the sensor body. Hence, for V3, a completely new design of the sensor body was developed specifically to improve the bending torque measurements. This is achieved by separating the axial from the bending sensitivity. V3 shows the lowest errors in the bending direction.

For ease of comparison between the sensor-integrating bolt iterations, plots were generated for visualization: Figure 13 shows the maximum of linearity and hysteresis error (e_lin_, e_hyst_) with respect to the stress increase for axial and bending excitation. Again, the conflict of objectives is notable between a good mechanical versus a good sensory performance in the axial direction for V1, V2.2, and V3. In general, V3 is the best solution, especially for bending torque measurements, because it has a very low error in that regard. This comes with the cost of lower mechanical performance, although the von Mises stress is about 24% higher.

## 4. Discussion

The research question “How do evolving iterations of sensor-integrating bolts perform across key mechanical and sensory characteristics?” can be addressed on the basis of the obtained results. Across successive iterations, several aspects of the sensor-integrating bolts were continuously improved, including the uncertainty associated with strain gauge application, measurement errors, sensitivity, and the accuracy of bending torque measurement. Sensory performance is evaluated through linearity error, hysteresis error, and sensitivity, whereas mechanical performance is assessed by the increase in von Mises stress and the remaining percentage of the minimum cross-sectional area compared to a full bolt. Figure 13 illustrates a clear trade-off between mechanical and sensory performance. Enhancing sensory performance leads to a reduction in mechanical performance, highlighting the inherent conflict of objectives when aiming for a high degree of fulfillment in both mechanical and sensory function.

**Influence of sensory function on mechanical performance**: Overall, sensor integration alters the mechanical characteristics of the bolt to a measurable extent. The stress increase ranges from 1% to 24% compared to the unchanged reference bolt (see Table 3, Figure 13). Comparable effects on mechanical performance are reported for state-of-the-art sensor-integrating bolts, where the sensor is embedded directly into the bolt structure. For instance, Groche et al. [21] report a reduction in load-carrying capacity of approximately 20%. To compensate for this loss in loadability, appropriate measures must be implemented, such as increasing the available material by selecting a higher strength class.

**Sensory performance**: Comparisons with state-of-the-art multi-axis force sensors based on strain gauges with comparable force ranges [17,46,47] are conducted in this section. It should be noted that sensor-system solutions are very individual, therefore the setups and test conditions may vary, which has to be considered when comparing absolute values. The state of the art for multi-axial force sensors based on strain gauges with similar measurement ranges shows performances in a similar range (Table 4). It has to be noted that those solutions use more space for their strain gauge setup, which might be a reason for slightly better performance.

The similarity between TC2 (tightening) and TC1 (axial excitation) aligns with the state of the art: the torsional stress is reduced, for the most part, shortly after setting the bolt during assembly [7,38], therefore playing an insignificant role in the long-term operation of the bolt and also in the need to measure it.

Furthermore, the current characterization of sensory performance is limited to linearity error, hysteresis error, and sensitivity. For multi-axial force sensors, cross-talk between measurement axes represents an additional relevant performance metric. This effect can be quantified using a decoupling matrix to compute the multi-axis load vector from the sensor signals. The investigation of cross-talk and the derivation of such a decoupling matrix are subjects for future work.

Achieving high sensory performance requires sufficient design freedom in terms of available space for the sensor module. This design freedom enables the development of concepts such as the three-bending-beam configuration implemented in variant V3, as well as the integration of electronic components for signal sampling and data transmission, as presented by Riehl et al. [34,42]. This increased allocation of space for the sensory function results in a reduced availability of space for the mechanical function, as reflected by the fraction of the minimum cross-sectional area (Table 3, Figure 13).

Variant V3 represents the most suitable solution, particularly for bending torque measurements, as it exhibits very low measurement error. The miniaturized F^2^LECS electronics have proven to be effective in acquiring and transmitting sensor signals while maintaining low space requirements and low power consumption. Consequently, this approach offers a significant advantage for many state-of-the-art sensor-integrating bolts, which often lack full integration of the complete measurement chain due to limitations in available space and power. Long-term stability and suitability for industrial operation conditions have not been demonstrated yet and will be tested in future work.

**Apparent superposed bending torque on axial excitation**: The results indicate that the sensitivity of one strain gauge is higher than that of the other two, suggesting the presence of a superposed bending torque despite the purely axial excitation. This behavior may be attributed either to issues during strain gauge application or to manufacturing tolerances and surface irregularities within the universal testing machine setup. However, strain gauge application effects as the primary cause can be excluded, as the observed sensitivity pattern changes when the bolt is rotated relative to the clamped components.

**Position of the strain gauges for bending measurements**: With respect to the axial position, all design iterations place the strain gauges within the bolt shaft, where an approximately homogeneous strain distribution is present [32]. Regarding the radial position relative to the central axis, the results demonstrate that strain gauge sensitivity increases with increasing radius (Figure 8). This observation is consistent with the fundamental physical principle governing strain distribution in bodies subjected to bending, where strain magnitude increases with distance from the neutral axis [48].

**Evolving from failing iteration V2.1 to V2.2**: An interesting observation to highlight is the transition from V2.1 to V2.2. The failure of the interference fit in V2.1 was addressed by deliberately weakening the sensor body through the introduction of a borehole, thereby reducing its cross-sectional area and the force acting upon it. Increasing the interference fit itself was not feasible, as this would have risked damaging the mating surface. While the weakening of the sensor body leads to a reduced minimum cross section and consequently lower load-carrying capacity, it simultaneously results in increased sensitivity (Table 3). This trade-off once again highlights the inherent conflict of objectives between mechanical robustness and sensory performance.

## 5. Conclusions

This contribution advances the state of the art by presenting a comprehensive characterization of evolving iterations of sensor-integrated bolts for combined axial force and bending torque measurement, featuring a fully integrable measurement chain with a BLE interface. The detailed insights gained from each iteration are systematically documented, highlighting the central role of experimental testing in the methodical development of sensor-integrating bolts. The study provides valuable insights into how sensor placement and installation strategies influence signal quality and overall system performance, as well as into the inherent conflict of objectives between mechanical and sensory function. By embedding three half-bridge strain gauges in different configurations within an M20 bolt, together with the miniaturized F^2^LECS electronics for strain gauge signal acquisition and wireless communication, the proposed concept enables the in situ measurement of axial forces and bending torque vectors without compromising the standardized mechanical interfaces of the bolt.

The results demonstrate that the strain gauge signals reliably follow the applied loads, exhibiting linear and repeatable behavior over multiple axial and bending load cycles. The sensory characterization yields performance errors below 6% and sensitivities of approximately 10 µV/V/kN for axial loading and 1 µV/V/Nm for bending moments. Under ideal conditions, this corresponds to a resolution of 6 N for axial force measurement and 46 mNm for bending torque measurement alone. The bending resolution of variant V3 will be investigated in future work. The mechanical performance is reduced, shown with respect to the two most common failure modes of (1) increased stress (1.26% to 24.6%), and (2) reduced cross section (97% to 70%), depending on the specific sensor concept and its associated space requirements.

Furthermore, the comparison of the different iterations highlights the inherent conflicts of objectives: enhanced sensory performance is achieved at the expense of reduced mechanical performance. Integrating sensors into load-bearing machine elements inevitably alters their mechanical properties, which must be compensated through appropriate design measures, for example, by material selection.

In future work, model-based approaches will be implemented to calculate axial forces and bending torque vectors. This will introduce an additional source of error that will affect all iterations in a comparable manner. Moreover, temperature dependence as well as dynamic and long-term behavior will be investigated. Another open research topic is the measurement of combined axial force and bending torque excitations to enable a quantitative assessment of cross-talk effects.

## Figures and Tables

**Table 2:** Test cases mapped to the characteristics and versions of sensor-integrating bolts. Test cases are measured with state-of-the-art strain gauge amplifier (GSV4) and miniaturized electronics (F^2^LECS).

Test Case	Characteristics	Vi	Max. Excitation
1. Central axial load (tensile)	Axial: e_Lin_, e_Hyst_, s	GSV4/8 and F^2^LECS	50 kN
2. Tightening (tensile+shear)	Axial: e_Lin_, s	GSV4	200 Nm tightening torque wrench
3. Three-point bending	Bending: e_Lin_, e_Hyst_, s	GSV4	200 Nm bending torque

**Table 3:** Characteristics of the sensor-integrating bolts, iterations V1–V3.

	Characteristics	V1	V2.1	V2.2 (GSV4)	V2.2 (F^2^LECS)	V3 [35]
Sensory axial (TC1)	max(e_lin,SG1–3_) in %	3.30	27.34	2.20	4.85	1.01 [35]
	max(e_hyst,SG1–3_) in %	4.00	54.00	2.21	6.02	6.15 [35]
	Mean(sens_SG1–3_) in µV/V/kN	10	4	9.8	9.9	5
Sensory axial (TC2)	max(e_lin,SG1–3_) in %	3.73	17.89	2.05	-	-
	Mean(sens_SG1–3_) in µV/V/kN	10.6	4.5	9.5	-	-
Sensor bending	max(e_lin,SG1–3_) in %	3.68	-	3.67	-	0.11 ^1^
	max(e_hyst,SG1–3_) in %	2.53	-	2.73	-	0.52 ^1^
	Mean(sens_SG1–3_) in µV/V/Nm	1.3	-	0.9	-	-
Mechanical	Relative v. Mises stress increase (FEM) in %	1.26	18.4 [32]	18.4 [32]	18.4 [32]	24.6 [32] 23.5 [35]
	Percentage of minimal cross section of full bolt in %	97	91	73	73	70

^1^ Only sensor body, not mounted in bolt.

**Table 4:** Comparison of sensory performance of the state of the art. The cited sources may have dynamic testing as well, although here, only the published static testing is compared.

Source	Sensor Principle	Measurement Range	Test Conditions	Linearity Error	Hysteresis Error	Sensitivity
V2.2 GSV4	3-axis: Deformation body using 3 Wheatstone half-bridges	0 to 50 kN 0 to 200 Nm	Static uniaxial loading in universal testing machine	Axial: 2.2% Bending: 3.7%	Axial: 2.2% Bending: 2.7%	Axial: 9.8 µV/V/kN Bending: 0.9 µV/V/Nm
Feng et al. [46]	6-axis: Self-decoupled cross beam structure using 6 full Wheatstone bridges	±120 kN ±30,000 Nm	Static uniaxial loading in wheel test assembly	Axial X: 2.4% Bending (x,y max): 1.7%	Axial: 1.3% Bending: 1.4%	Axial: 10.5 µV/V/kN Bending: 0.0012 µV/V/Nm
Lin et al. [47]	3-axis wheel force transducer	0 to 14 kN 0 to 3000 Nm	Static loading	0.6–0.9%	0.9–1.1%	No data

**Figure A1:**
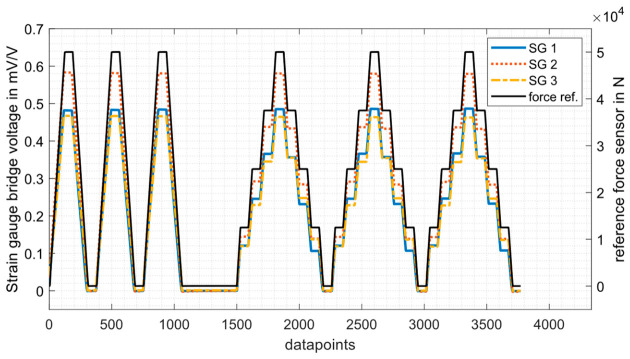
Excitation cycle of the central axial load case for V1. The strain gauge signals follow the reference very well, but deviate slightly from each other, indicating a bending torque. The data record frequency is 12.5 Hz.

**Figure A2:**
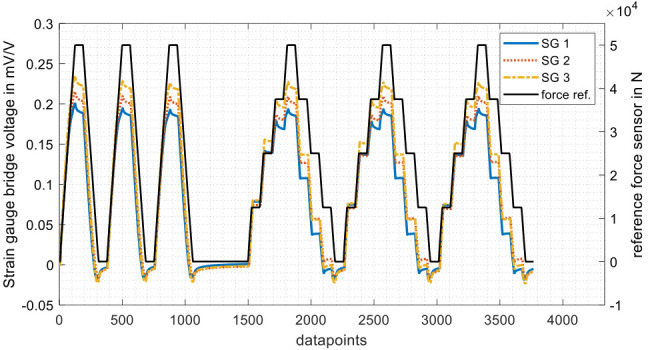
Excitation cycle of the central axial load case for V2.1. The SG signals above an excitation of approx. 20 kN are not constant during constant load, but decrease over time; this also happens in the downstepping at loads of 0 N. The data record frequency is 12.5 Hz.

**Figure A3:**
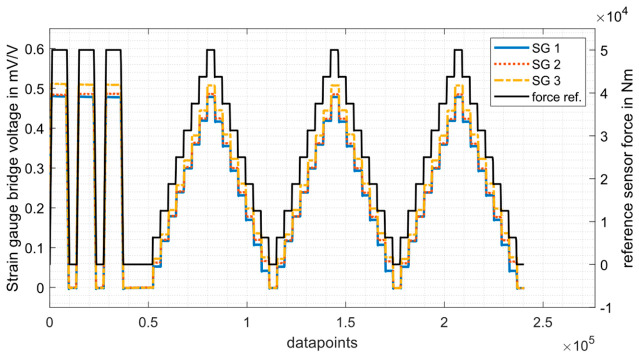
Excitation cycle of the central axial load case for V2.2. The strain gauge signals follow the reference very well. The data record frequency is 100 Hz.

**Figure A4:**
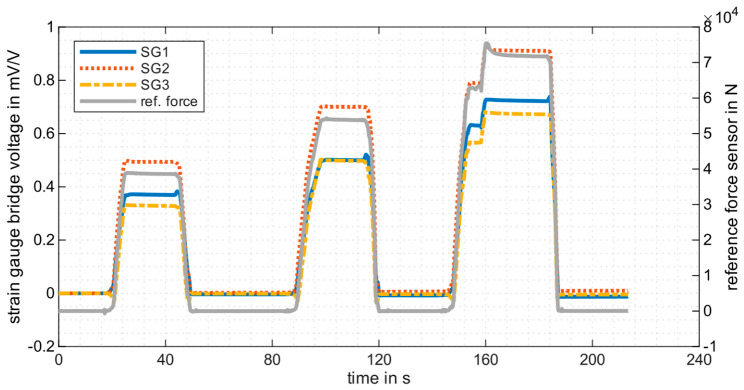
Timeseries plot of tightening and loosening for V1, with 100 Nm, 150 Nm and 200 Nm of tightening torque showing superposition of bending torques, due to spacing of signal levels and return to zero after each loosening.

**Figure A5:**
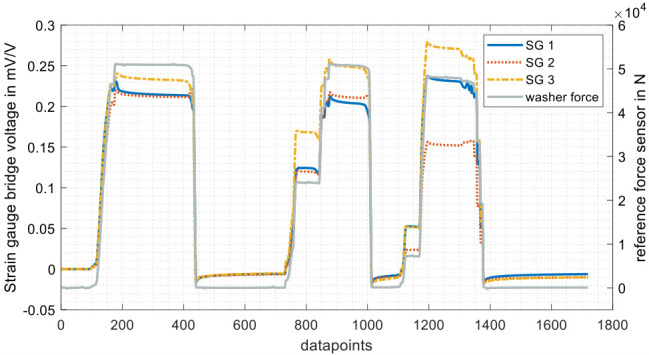
Timeseries plot of tightening and loosening for V2.1; superposed bending torques of varying degree are apparent; SG signals decrease slightly with constant reference force, hinting at a defect of the sensor body’s interference fit or strain gauge application.

**Figure A6:**
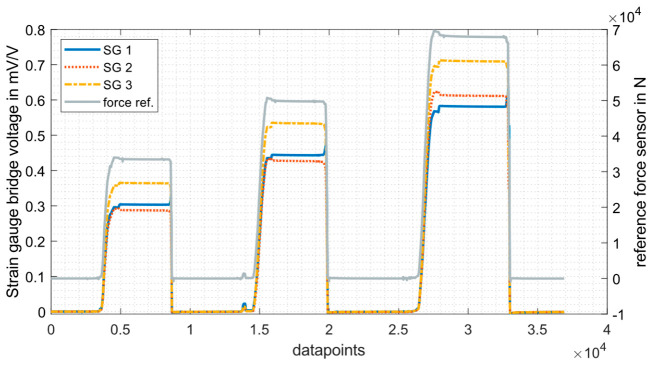
Timeseries plot of tightening and loosening for V2.2, with 100 Nm, 150 Nm and 200 Nm of tightening torque showing superposition of bending torques, due to spacing of signal levels and return to zero after each loosening.

**Figure A7:**
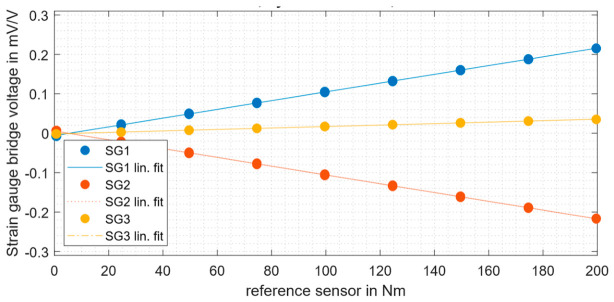
Linear fit plot for excitation direction 331° with respect to SG1 showing low errors below 2.2% and individual sensitivities per SG due to the bending strain field in the bolt.

**Figure A8:**
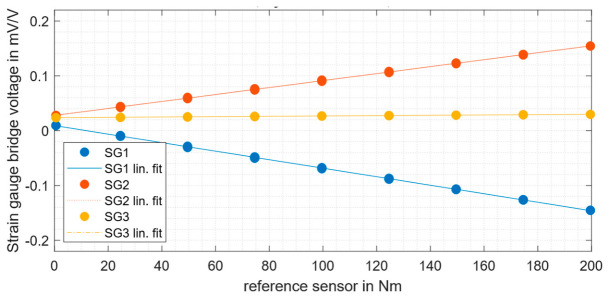
Linear fit plot of test case 3: three-point bending of V2.2 with excitation from direction 153° with respect to SG1.

## Data Availability

The original data generated for this article are publicly available in the repository KITopen ID: 1000192383, https://doi.org/10.35097/983cwbqtmcmzqahw.

## Abbreviations and Symbols

The following abbreviations are used in this manuscript:

SG	Strain gauge
BLE	Bluetooth Low Energy
NFC	Near-field communication
ADC	Analog-to-digital converter
ENOB	Effective number of bits
AFE	Analog front end
ADS1220	AFE from Texas Instruments used in the miniaturized electronics for acquiring SG signals
F^2^LECS	Flexible Low-Power Embedded Circuit for Strain Gauges in Sensor-Integrating Machine Elements
GSV4/8	State-of-the-art strain gauge amplifier and digitization system from ME-Systeme
SiME	Sensor-integrating machine element
S	Sensitivity
fit	Linear fit of datapoints
sensor_i_	One measurement datapoint of sensor element
F	Force
σ	Stress
A_min_	Minimal cross section
A_ref_	Cross section at shaft of an unchanged reference bolt according to ISO 4014
ε	Strain
E	Young’s modulus
e_lin_	Linearity error
e_hyst_	Hysteresis error

## Appendix A

### Appendix A.1. Additional Data and Plots Regarding Test Case 1: Central Axial Excitation

The strain gauge signals follow the reference force sensor very well in a repeatable pattern over the three load cycles, returning to 0 after unloading. Concerning V2.1, above a load of approx. 20 kN, the SG values are not constant during constant load, but decrease over time resembling an exponential function, as observed previously during tightening. It is assumed that the interference fit fails and the sensor body does not follow the strain.

**Table A1:** Test case 1 central axial excitation: criteria, characterizing sensory performance.

SG#	Criteria	V1	V2.1	V2.2 (GSV4)	V2.2 (ADS1220)
SG1	e_lin_	2.29%	27.34%	2.20%	2.23%
	e_hyst_	2.96%	54.00%	2.21%	2.27%
	s in mV/V/kN	0.0098	0.0040	0.0097	0.0102
SG2	e_lin_	0.86%	20.28%	1.46%	1.72%
	e_hyst_	1.37%	39.63%	0.91%	2.64%
	s in mV/V/kN	0.0117	0.0043	0.0097	0.0102
SG3	e_lin_	3.30%	22.25%	1.76%	4.85%
	e_hyst_	4.00%	43.71%	0.81%	6.02%
	s in mV/V/kN	0.0092	0.0048	0.0100	0.0094
SG1..3	max(e_lin,SG1–3_)	3.30%	27.34%	2.20%	4.85%
	max (e_hyst,SG1–3_)	4.00%	54.00%	2.21%	6.02%
	Mean(sens_SG1–3_) in mV/V/kN	0.010	0.004	0.0098	0.0099

### Appendix A.3. Additional Data and Plots Regarding Test Case 3: 3-Point Bending

From the linear fit plot (Figure A7), the sensitivities and errors for each SG are calculated.

**Table A2:** Test case 3: 3-point bending excitation: criteria characterizing sensory performance, mean errors and max sensitivities of all angles.

SG#	Criteria	V1	V2.2
SG1	e_lin,mean_	1.30%	2.42%
	e_hyst,mean_	1.02%	2.41%
	sens_max_ in µV/V/Nm	1.2	0.90
SG2	e_lin,mean_	3.68%	3.67%
	e_hyst,mean_	2.53%	2.73%
	sens_max_ in µV/V/Nm	1.35	0.91
SG3	e_lin,mean_	1.66%	1.21%
	e_hyst,mean_	1.50%	0.84%
	sens_max_ in µV/V/Nm	1.45	0.88
SG1..3	max(e_lin_)	3.68%	3.67%
	max(e_hyst_)	2.53%	2.73%
	mean(s) in µV/V/Nm	1.3	0.9

## References

[1] Vorwerk-Handing G., Gwosch T., Schork S., Kirchner E., Matthiesen S. (2020). Classification and examples of next generation machine elements. Forsch. Ingenieurwes.

[2] Kirchner E., Wallmersperger T., Gwosch T., Menning J.D.M., Peters J., Breimann R., Kraus B., Welzbacher P., Küchenhof J., Krause D. (2023). A Review on Sensor-integrating Machine Elements. Adv. Sens. Res..

[3] Brenneis M., Groche P. (2014). Integration of Piezoceramic Tube under Prestress into a Load Carrying Structure. Adv. Mater. Res..

[4] (1999). ISO General Purpose Metric Screw Threads—Part 1: Nominal Sizes for Coarse Pitch Threads; Nominal Diameter from 1 mm to 68 mm.

[5] (2022). Fasteners—Hexagon Head Bolts—Product Grades A and B.

[6] (2013). Mechanical Properties of Fasteners Made of Carbon Steel and Alloy Steel—Part 1: Bolts, Screws and Studs with Specified Property Classes—Coarse Thread and Fine Pitch Thread (ISO 898-1:2013).

[7] (2015). Systematic Calculation of Highly Stressed Bolted Joints, JOINTS with One Cylindrical Bolt.

[8] Rousseau R.I., Bouzid A.-H. (2024). The Tightening and Untightening Modeling and Simulation of Bolted Joints. Machines.

[9] Blachowski B., Gutkowski W. (2016). Effect of damaged circular flange-bolted connections on behaviour of tall towers, modelled by multilevel substructuring. Eng. Struct..

[10] Abid M., Nash D.H. (2006). Bolt Bending Behaviour in a Bolted Flanged Pipe Joint: A Comparative Study. ASME 2006 Pressure Vessels and Piping/ICPVT-11 Conference, Vancouver, BC, Canada, 23–27 July 2006.

[11] Wiegand H., Kloos K.H., Thomala W. (1988). Tragfähigkeit von Schraubenverbindungen bei mechanischer Beanspruchung. Schraubenverbindungen.

[12] Rappenecker K., Peinhardt F., Geiser F., Peters J., Kachel G., Isele A., Matthiesen S., VDI Wissensforum GmbH (2025). Vorstellung einer sensorintegrierten Schraube zur Bestimmung der Gesamtzusatzspannung. Schraubenverbindungen, Berechnung, Gestaltung, Montage, Anwendung, Leipzig, 12–13 November 2025.

[13] Fraunhofer Institute for Surface Engineering and Thin Films IST Smart Screw Connection—Wireless and Energy-Autonomous Monitoring Solution. https://www.ist.fraunhofer.de/content/dam/ist/de/documents/jb/2022/jb2022_de_intelligente-schraubverbindung-qbo.pdf.

[14] Biehl S., Paetsch N., Meyer-Kornblum E., Bräuer G. (2016). Wear resistenat thin film sensor system for industrial applications. Int. J. Instrum. Meas..

[15] Baki P., Székely G., Kósa G. (2013). Design and characterization of a novel, robust, tri-axial force sensor. Sens. Actuators A Phys..

[16] Schuster A., Otto A., Rentzsch H., Ihlenfeldt S. (2024). Multi-Sensory Tool Holder for Process Force Monitoring and Chatter Detection in Milling. Sensors.

[17] Templeman J.O., Sheil B.B., Sun T. (2020). Multi-axis force sensors: A state-of-the-art review. Sens. Actuators A Phys..

[18] Frank T., Grün A., Kermann M., Cyriax A., Steinke A., Ortlepp T., Reschke G. (2019). Hybridintegration von Mikrodehnungssensoren (Hybrid integration of micro strain sensors). 20. GMA/ITG-Fachtagung Sensoren und Messsysteme 2019.

[19] Wang T., Zhang W., Yang D., Wang H., Lu G., Lu M. (2024). Head surface strain measurement based wireless bolt sensor with self temperature compensating. Meas. Sci. Technol..

[20] Sanli A., Demirkale B., Kanoun O. (2025). Real-time detection of loosening torque in bolted joints using piezoresistive pressure-sensitive layer based on multi-walled carbon nanotubes reinforced epoxy nanocomposites. Sci. Rep..

[21] Groche P., Brenneis M. (2014). Manufacturing and use of novel sensoric fasteners for monitoring forming processes. Measurement.

[22] ConSenses GmbH Assembly Instructions and Datasheet PiezoBolt. https://consenses.de/fileadmin/user_upload/Assembly_Instructions_and_Datasheet_PiezoBolt.pdf.

[23] Core Sensing GmbH coreIN Sensor Integration, 2024. https://core-sensing.de/en/produktgruppe/sensoren/corein/.

[24] Sensorise GmbH Sensorise SmartScrew. https://sensorise.de/solutions/smartscrew/.

[25] VALLEY FORGE and BOLT SPC4 Load Indicating System Product Information. https://www.vfbolts.com/product/spc4-load-indicating-system-2/.

[26] Intellifast Intellifast System—Intellifast. https://www.intellifast.de/intellifast-system/.

[27] Guo Y., Hu Z., Xiong L., Zhou X., Zhu P. (2022). Fiber Bragg grating based quasi-distributed bolt force sensor with torque resistance. Measurement.

[28] Li T., Liu W., Gao H., Wang N., Xia K., Li R., Tan Y., Zhou Z. (2024). FBG-based force sensing with temperature self-compensation for smart bolts. Sens. Actuators A Phys..

[29] Horn S., Gwosch T., Matthiesen S. (2021). Sensor-integrated chemical anchor for continuous monitoring of the fastening situation. Beton-und Stahlbetonbau.

[30] German Research Foundation DFG Priority Programme 2305: Sensor Integrating Machine Elements: Enablers for Comprehensive Digitization. https://www.spp2305.de/.

[31] Dumstorff G., Paul S., Lang W. (2014). Integration Without Disruption: The Basic Challenge of Sensor Integration. IEEE Sens. J..

[32] Peters J., Mirbach N., Herbst F., Riehl D., Kupnik M., Hofmann K., Matthiesen S. (2024). Overcoming Conflicts of Objectives between Sensory and Mechanical Domain in the Development of Sensor-Integrating Machine Elements Using the Example of Bolts. IEEE Access.

[33] Peters J., Doellken M., Grauberger P., Herbst F., Riehl D., Kupnik M., Hofmann K., Matthiesen S. (2025). Design Support and Strategies for Integrating Sensing Functions into Machine Elements (in press). Procedia CIRP.

[34] Riehl D., Korner D., Keil F., Peters J., Matthiesen S., Hofmann K. (2024). Flexible Modular Electronic Platform for Sensor-Integrating Bolts. EBL 2024: Elektronische Baugruppen und Leiterplatten.

[35] Herbst F., Chadda R., Peters J., Riehl D., Hartmann C., Suppelt S., Breimann R., Kirchner E., Hofmann K., Matthiesen S. (2024). Sensor-Integrating Bolt for Multiaxial Force Measurement. IEEE Sens. J..

[36] (2021). Development of Mechatronic and Cyber-Physical Systems.

[37] Ştefǎnescu D.M. (2011). Handbook of Force Transducers: Principles and Components.

[38] Wiegand H., Kloos K.-H., Thomala W. (2007). Schraubenverbindungen: Grundlagen, Berechnung, Eigenschaften, Handhabung, 5.

[39] Bonaiti L., Knoll E., Otto M., Gorla C., Stahl K. (2022). The Effect of Sensor Integration on the Load Carrying Capacity of Gears. Machines.

[40] (2011). Metallic Materials—Calibration of Force-Proving Instruments Used for the Verification of Uniaxial Testing Machines.

[41] (2021). Metallic materials—Bend test (ISO 7438:2020).

[42] Riehl D., Herbst F., Peters J., Leiacker D., Matthiesen S., Kupnik M., Hofmann K. (2025). Flexible Ultra-Low Power Strain Gauge Readout Platform for Sensor-Integrating Bolts. 2025 IEEE International Instrumentation and Measurement Technology Conference (I2MTC), Chemnitz, Germany, 19–22 May 2025.

[43] Herbst F., Chadda R., Hartmann C., Peters J., Riehl D., Gwosch T., Hofmann K., Matthiesen S., Kupnik M. (2022). Multi-axis Force Sensor for Sensor-integrating Bolts. 2022 IEEE Sensors, Dallas, TX, USA, 30 October–2 November 2022.

[44] Noh Y., Bimbo J., Sareh S., Wurdemann H., Fraś J., Chathuranga D.S., Liu H., Housden J., Althoefer K., Rhode K. (2016). Multi-Axis Force/Torque Sensor Based on Simply-Supported Beam and Optoelectronics. Sensors.

[45] Budynas R.G., Nisbett K.J. (2015). Shigley’s Mechanical Engineering Design.

[46] Feng L., Lin G., Zhang W., Pang H., Wang T. (2015). Design and optimization of a self-decoupled six-axis wheel force transducer for a heavy truck. Proc. Inst. Mech. Eng. Part D J. Automob. Eng..

[47] Lin G., Pang H., Zhang W., Wang D., Feng L. (2014). A self-decoupled three-axis force sensor for measuring the wheel force. Proc. Inst. Mech. Eng. Part D J. Automob. Eng..

[48] Böge A., Böge W. (2024). Formeln und Tabellen zur Technischen Mechanik.

